# How does the experience of the medical encounter with Mayer-Rokitansky-Küster-Hauser (MRKH) syndrome impact women in Africa?

**DOI:** 10.11604/pamj.2022.42.314.32830

**Published:** 2022-08-26

**Authors:** Rose-Danielle Ngoumou

**Affiliations:** 1 University of Yaounde 1, Department of Sociology, Yaounde, Cameroon

**Keywords:** Women, Mayer-Rokitansky-Küster-Hauser (MRKH) syndrome, medical encounter, Africa

## Abstract

**Introduction:**

the MRKH syndrome is a rare congenital malformation in women. As most rare conditions, this syndrome usually causes confusion in patients and even in physicians. The medical encounter of young girls with the syndrome is intertwined in a complex matrix of social, economic and cultural factors that only contribute in creating more confusion in the young girl.

**Methods:**

the study is qualitative and designed to describe the first encounter of women with the MRKH syndrome at the physician. To that effect, an interview guide was designed for in-depth interviews with 05 MRKH women from varied African countries (Cameroon, Côte-d’Ivoire, and Senegal) selected using the snowball technique.

**Results:**

findings from this study revealed that most women go to the hospital and return more confused as no clear information is given to them on their condition. Also, cultural, social and religious beliefs on the one hand seems to blur the physician’s judgment to provide appropriate remedies like instrumental dilatation for rudimentary vagina, surrogacy for uterine infertility factor, adoption, etc. and on the other hand, these beliefs also act as a barrier for these young girls who are not strangers to the culture.

**Conclusion:**

the study suggests that, physicians give to the patients all necessary information for them to take elective decisions on their health. It also suggests that awareness should be raised on this condition.

## Introduction

The Mayer-Rokitansky-Küster-Hauser (MRKH) syndrome is not very much documented in most African countries. In Cameroon, a few studies have focused on creating awareness on the existence of this syndrome and giving orientations on how such a case can be handled [1], others have depicted the experience of such women in suburban areas while showing the psychological distress these women go through [2]. Also known as Mullerian agenesis, this syndrome is the situation where the Mullerian canals responsible for the formation of the uterus and the vagina fail to develop at the 6^th^ month of embryogenesis, thereby causing a girl to be born with either one of these organs or none at all [3]. The MRKH syndrome is a rare malformation that affects 1/4500 women globally [4]. It is estimated to be one of the major causes of gynecological consultations in France [5]. The *«Mayer-Rokitansky-Küster-Hauser (MRKH) syndrome, also referred to as Müllerian agenesis, is the second most common cause of primary amenorrhhoea. This disorder belongs to the most severe uterine malformation category (class 5) of the European Society of Human Reproduction and Embryology/European Society for Gynecological Endoscopy (ESHRE/ESGE) classification. It is characterized by congenital absence of the uterus, cervix, and upper part of the vagina in otherwise phenotypically normal 46, XX females»* [6]. Women with the MRKH syndrome therefore will never have their periods nor have children of their own. This syndrome is only diagnosed at the stage of puberty with a retard in the onset of menstruation. *«In general, the diagnosis will be made during adolescence when a woman with otherwise typical growth and pubertal development presents with primary amenorrhhoea»* [7]. This is the most troubling and distressing fact about the MRKH syndrome. This is because the age of menstruation coincides with a very delicate time of the girl child´s development; puberty. In fact, puberty is a transitional phase from childhood to adulthood wherein a girl may experience the changes in her body with mixed feelings.

The present study aims to explore the experiences of patients suffering from the MRKH syndrome in the interaction with their physician. The first encounter at the physician for the MRKH syndrome is always a surprise as the motive of consultation is always to understand the reason for primary amenorrhhoea. The physician even in the absence of specific expertise should be able to deliver a discourse that can contribute to the mental health of the patient as *«Young women diagnosed with MRKH syndrome suffer from extreme anxiety and very high psychological distress when they are told they have no uterus and vagina. Thus, it is recommended that the patient and family should be counselled before and throughout treatment»* [8]. The diagnosis can be a stepping stone to many hardships that can modify the life of these young women. In the face of this complex syndrome physicians have to make extra efforts to not project their beliefs or expectations onto the patient. It is therefore important to associate various specialists for a holistic care of these young women after they recieve their diagnosis because: *«Adjusting to the diagnosis of MRKH is a difficult and traumatic process for these women, leading them to question their identity as women and to experience a sense of confusion regarding their gender, their bodies, and their social and sexual roles. This threat gives rise to negative self-beliefs, with many women seeing themselves as defective, inferior, or unloveable (…) their infertility, in particular, may serve to perpetuate these women´s defective sense of self»* [9]. Thus, the present study envisages to show what impacts the medical encounter of women with the MRKH syndrome have in their lives. The scarcity of information and the incapacity of physicians to give complete details about their condition is the starting point of numerous difficulties these young women face.

## Methods

The study used a qualitative inquiry method which allowed the researcher to explore in-depth descriptions, explanations, and narrations of the experiences of the respondents as regards their first encounter at the physician. Five (05) MRKH women from three (03) African countries (Cameroon, Côte-d´Ivoire, and Senegal) selected using the snowball technique participated in this study. We got the contact of one woman in Cameroon with MRKH syndrome who introduced us to a WhatsApp group made of 10 MRKH women from different countries. Informed consent to take part in the study was obtained from each participant. Only those who gave their consent to participate in the study were included. The researcher disclosed the content of the consent notice to the participants to inform them that their participation in the study was voluntary, therefore they could withdraw at any time during the research. The researcher, conducted the interviews using a voice recorder with participants in Cameroon where permission was secured. Participants out of Cameroon were asked to respond by voice notes on WhatsApp. The different participants were attributed codes in the place of their real name. Our informants all responded in French, and their verbatim were translated to English for the purpose of this paper.

## Results

**Sociodemographic profil of respondents:** we interviewed a total number of five (05) women and their ages ranged between 23 and 30 years. All participants have completed secondary education and are enrolled in a university program. Their university degrees included bachelor´s to master´s degree. We had two (02) catholics, two (02) muslims and one (01) pentecotist in the study. The age interval of diagnosis was between 15 to 24 years. All were diagnosed with MRKH syndrome with vaginal and uterine agenesis but for two who also had absent ovaries.

**The «scarcity» of information:** the majority of women with Mayer-Rokitansky-Küster-Hauser said they were not given enough information to help them understand and cope with their condition. They went to the hospital and returned more confused as they had a number of unanswered questions that kept on swirling their mind. A respondent from Senegal tells us that: *«I was diagnosed in 2014, at the time I was 15 years. I went to the hospital because I did not see my menses. I was asked to do a pelvic ultrasound and from there I was told I have a congenital malformation. Then i was asked to do an MRI to confirm these results. The MRI showed the absence of vagina, vaginal and uterine agenesis. That was what the MRI showed and the doctor didn´t give me any more informations so I didn´t know the name of this syndrome»* SEN1. The MRKH syndrome is unknown by many in society. It is a syndrome that diminishes a woman´s self-esteem and covers her with shame. Therefore, most families choose to keep it secret in order to protect their relative from the trauma that can arise from disclosing the condition to non-relatives. This also makes it difficult to diffuse information on the condition. It makes information scarce and reduces the expertise of physicians on the syndrome, thereby making them «incapable» of giving clear information on the syndrome. The result of this is that most of these young women remain confused about what they are «suffering» from and can experience depression and develop negative thoughts about themselves. Another informant says: *«I was confused; I had never heard about this syndrome. There was nobody to relate about this, the doctors, my parents, nobody had heard about this before and had not seen anyone with the syndrome. I felt lost, completely»*CMR01. This exemplifies the major problem persons affected by a rare condition face. In fact there is a lack of information on rare diseases which points out the necessity to create more awareness on these diseases and also the need give them a platform.

**The imperative to become health literate: the «patient expert»:**this situation causes patients to develop their health literacy so they can understand and manage their condition properly. Most of the respondents reported they learnt to be knowledgeable about their condition. This is important as it helps them cope with the stress linked to their condition hence reinforcing their resilience. Thus, the burden of psychological distress is reduced in the process. This is the exemple of SEN 1 who after spending two years in total ignorance of her condition decided to search for the information herself: *«In 2016 I started taking my diagnosis seriously, i started looking for information on the internet and consulting gynecologists but unfortunately till today no gynecologist has helped me. I am still at the same level only that I have learned to better understand the syndrome and its remedies»* SEN1. Globally, all informants reported they had not recieved clear information about their condition at the hospital and because of that they had to develop strategies that could help them have an insight into what they were suffering from. In fact, one respondent says, talking about the physician she met: *«He didn´t really have the occasion nor the time to explain things in details. I got all the information I know about the condition myself using the terms that were written on my exam bulletin, ultrasound report. But i also came along persons who thought they knew what i was suffering from because in reality no gynecologist talked about MRKH. All were talking about an imperforated vagina»*CI01. The insctinct to «survive» in these women pushes them to become health literate and in the process enables them to better understand what is «lacking» in them.

**The extensions of the MRKH syndrome:**the MRKH syndrome is rare and families usually do not have any information on it until they have a case of MRKH syndrome. Women of our study reported they felt lonely and alone in their battle because they did not recieve any support from their family. A few told us they had to struggle all alone to understand what they were actually facing. Furthermore, they had to think out «survival» strategies to cope as they felt alone and abandonned in their journey with the MRKH syndrome. It is the case of this informant who says that: *«I went to the gynecologist because honestly our situation, is abandonment of parents that tire us in Africa when the thing is discovered, humm, how can I say this, we have the impression that it does not bother parents, or parents, they classify us in a certain position, they dont really care about us, that´s it. They dont really care about us»*CIO1. From this informant’s response, we can clearly see a troubled mind, given the repetition of certain words such as: *«we have the impression that it does not bother parents or parents, they classify us in a certain position, they dont really care about us, that’s it. They dont really care about us»* which in fact serves to lay emphasis on what is said. Due to this situation which is relevent of the confusion that rare conditions can cause, most of these young girls are obliged to open up to people neutral. *«I talked about it given that it is the social affairs service of my school that did my first consultation because there is a lady there who works with the social service of my school to whom i talked to, I asked her if it was normal and it was her, i can she was the one who opened my medical record»* CIO1. Our informants told us they sometimes craved to talk to their families about their deep feelings regarding their condition. For some it is difficult to discuss such matters with their family because they feel ashamed or are simply not considered by their families. Families of these geographic areas give more value to a woman when she can bear children and cause a man to marry her. A woman who cannot carry out this function is simply regarded as a failure. As a result of this, they sometimes prefer to confide in anyone who shows them some empathy or compassion.

**The first encounter:** the first encounter at the hospital is very determining in the way these young women will live their condition. Given the complexe nature of the syndrome, in the course of this encounter a number of factors get mixed up and causes either resistances or acceptance. Therefore, one informant recalls: *«I was not satisfied about my first consultation because, hmmm, the main purpose of the doctor was to find out why I did not have my periods so, while he was carrying the gynecological examination and he noticed that may be there was no uterus, he sent me to do further investigations, after that, humm they found out I had no uterus and the other organs were functional, humm we returned, to see the doctor, the said doctor, can you imagine, didn´t even know the name of the condition»* (CMR01). She further explains: *«After going for detailed investigations, when we came back to see the specialist who interpreted the ultra sound results that we had done, he interpreted them and then he said, the name of the disease he does not know hmmmm luckily that day I was in school, I had a class, I had a class, I was in school, I think I was still in secondary school, yes, I was in school and do you know what he proposed to my mother? He proposed scabrous solutions, that humm (silence), that they should ’take me from behind' imagine a doctor, imagine! It was, when, humm my mother told me, it was traumatising»* (CMR01). In the following statement when the informant says *«take me from behind»*, she is referring to anal sex. In her words she was proposed by her physician to practice anal sex since she had no vagina. Anal sex is a sexual practice that is very much attributed to homosexuals eventhough heterosexual couples sometimes practice it out of fantasy despite the inconveniences that result from such a practice. If it is seen as a «normal» sexual practice by certain persons some people condemn the practice. The informant expresses her disapointement and see this as unorthodox.

The first encounter can also lead one straight to depression. It is therefore recommended that physicians use a pluridisciplinary approach if they can´t handle announcing the diagnosis. An informant tells us: *«The day the gynecologist put me in contact with a psychologist, I remember that I had to, hmmm , I went for an appointment, someone in my school to whom I had exposed my worry promised to go with at the gynecologist and also to know more about the condition to help me, so, at this appointment, the person did not come and so the gynecologist attended to me and then I asked him the question (laughter), if the surgery could atleast, hmmm, if after the surgery there were chances for me to conceive and (laughter) he had certain manners, he hmmm, he choosed the wrong manner to answer to my question, I was there, in front of him and he told me: «Are you dreaming or what? I told you there was nothing I could do at that level, all I can do is create a vaginal opening», I started crying in his office (laughter), I did not even ask him to call the psychologist»* (CI01). This shows that the patient-physician relationship is key to the « healing » process of patients and should therefore be improved on a daily basis.

**The confluence of cultural beliefs and the medical art:** the MRKH syndrome is a delicate condition as it touches the woman´s sexuality. Till today the woman has alot of restrictions as to what concerns her sexuality. We realized that a number of physician´s were equally influenced by these trends which draw their origins from sociocultural and religious beliefs. Vaginal dilatations are widely recognized as efficient methods for solving the problem of rudimentary vagina in MRKH women but this can be seen as masturbation which in some religious canons is viewed as sin. Moreover, religions that preach that women get into marriage while they are virgin are hardly receptive to vaginal dilation. In these belief systems, a good woman is one who does not have sexual relations outside marriage and with a man who is not her husband. Using vaginal dilatators takes away the “virginity” of the woman and can discredit her when she finally decides to get married. The Muslims are one of those religions that are rigorous about virginity at marriage. The words of this respondent who was a Muslim and was equally consulted by a muslim doctor clearly illustrates this: *«There was a gynecologist who talked to me about candles for dilatation but he told me to wait until my marriage is close. I think he does not want me to be a sexual vagabond. But I think it will be difficult to wait until marriage to start dilatation given that you need to do it for several months, regularly before reaching the length of 6 cm. My boyfriend even insisted on him doing it, he asked me to pay him some extra money so that he can agree to do the dilatations but the doctor still said no. I just told myself he knows nothing»* (SEN01).

Thus the dilatators raises a number of controversies to many African women who were raised knowing that virginity is a virtue and masturbation a vice. This can be seen in the following conception: *«He also proposed a type of thing, hmmm to enlarge my pelvis, I have to use an artificial penis, so I had to disvirgin myself with an artificial penis, I found this not… hmmm, I remember being a virgin when I was diagnosed, humm, I assure you I was traumatised, they will tell me I have to use an artificial penis, I simply said to myself that, hmmm even if I know a man it should happen naturally, even if I am disvirgined it should happen naturally not through these people´s methods»*(CMR01). According to this informant, sexual intercourse is a consensual activity and which should be done the «natural way». Practiced out of this nature, a number of moral questions arise. Therefore careful and detailed explanations should be given to patients on different treatment options so that they don´t feel their dignity or intimacy is being violated.

## Discussion

Cultural and religious beliefs have significant interferance with decision to choose a treatment option. Women seemed not to adhere to the practice of vaginal dilation either because it made them at risk of being sexually active before marriage or because it represented masturbation to them. The MRKH syndrome is a complexe condition and its impacts are felt at many levels of the life of women. Not only does it cause psychological distress, but it also alters the quality of life and self-esteem of the affected woman. Moreover, the medical encounter can also be the origin of profound depression and frustration. In fact, the physician, will have their own perceptions (representations of the syndrome), first of all, as an individual belonging to a space of sociability in daily life; that is, their subjectivity of the condition as an individual. Concerning the diagnosis, patients reported they left the hospital more confused and prone to depression. This situation was reported in the words of one informant who told us her physician not knowing what to do for her after the diagnosis proposed that she could practice anal sex to solve the problem of vaginal agenesis. This is why Turner´s view that: *«We can no longer regard diseases as natural events in the world which occur outside the language in which they are described. A disease entity is the product of medical discourses»* [10] is relevant. In MRKH syndrome standard treatment protocols recommend the creation of a neovagina or instrumental dilation of the vagina using dilators. A recent study on the issue supports the use of dilation therapy as the first-line treatment of rudimentary vagina and also emphasizes the relevance of coital dilation in patients able to regularly engage in coital activity [11]. The practice of anal sex is not adviced in this case as per the author´s knowledge and some authors have advanced that, increased rates of anal cancer may be attributable to more prevalent practice of anal intercourse [12].

In Africa, a woman´s value is deeply entwined and connected with reproductive and sexual expectations. The stigma surrounding infertility and sexual inactivity can magnify its emotional and physical consequences. Young women with the MRKH syndrome can lack support from their family as they are considered a shame. The young women of our study also deplored the lack of support from family. In their words, the condition causes even their own families to reject them and see them as inferior and “different“. This therefore justifies the view that, in the situation of a MRKH case: *«Family support plays an imperative role to console and raise the self-respect»* [13]. Families should be more supportive to help their relatives express all that they have within. They should be made to feel that their purpose in life is not reduced because of their condition.

## Conclusion

This research brings information about a relatively under researched area of young girls with the MRKH´s encounter at the physician in Africa. The study which aimed to comprehend how women affected by the MRKH syndrome experience the medical encounter and how this can have extensions to their participation in social life shows that it is intertwined in a complex matrix of social, economic and cultural factors that only contribute in creating more confusion in the young girl. The study shows that there is an urgent need to associate psychologists, counsellors or support groups to the treatment process of these patients. Furthermore, there is need to reinforce awareness raising campaigns on this syndrome and rare diseases in general.

### What is known about this topic

- The MRKH syndrome is a rare congenital malformation;
- The MRKH is a major cause of uterine infertility factor;
- The MRKH syndrome causes emotional and psychological distress in women.

### What this study adds

- This study assessed the distress young women diagnosed with the MRKH syndrome experience both in the hospital setting and in their families;
- The study contributes to raising awareness on the MRKH syndrome.

## References

[1] Egbe TO, Kobenge FM, Arlette MMJ, Nyemb JE, Mbu RE (2019). A case of Mayer-Rokitansky-Küster-Hauser syndrome in a low resource tertiary hospital in Douala, Cameroon. SAGE Open Med Case Rep.

[2] Ngoumou RD, Amada Talikoa, Nzhie Engono J (2020). Être femme faire face aux stigmates du syndrome de Mayer-Rokitansky-Küster-Hauser (MRKH) en milieu périurbain. (dir) Violences dans les sociétés contemporaines: constructions et vécus en Afrique Subsaharienne.

[3] Patnaik SS, Brazile B, Dandolu V, Ryan PL, Liao J (2015). Mayer-Rokitansky-Küster-Hauser (MRKH) syndrome: a historical perspective. Gene.

[4] Morcel K, Camborieux L (2007). Programme de Recherches sur les Aplasies Müllériennes (PRAM), Guerrier D. Mayer-Rokitansky-Küster-Hauser (MRKH) syndrome. Orphanet J Rare Dis.

[5] Reynaud S (2021). Syndrome de Mayer-Rokitansky-Kuster-Hauser. Thèse pour le Diplôme d´État de docteur en médecine. Limoges.

[6] Fontana L, Gentilin B, Fedele L, Gervasini C, Miozzo M (2017). Genetics of Mayer-Rokitansky-Küster-Hauser (MRKH) syndrome. Clin Genet.

[7] Weijenborg PTM, Kluivers KB, Dessens AB, Kate-Booij MJ, Both S (2019). Sexual functionning, sexual esteem, genital self-image and psychological and relationnal functionning in women with Mayer-Rokitansky-Küster-Hauser (MRKH) syndrome: a case-control study. Hum Reprod.

[8] Morten H, Bay Bjørn AM, Krogh Jørgensen L, Trolle B, Bjørn Petersen M (2018). Treatment of vaginal agenesis in Mayer-Rokitansky-Küster-Hauser syndrome in Denmark: a nationwide comparative study of anatomical outcome and complications. Fertil Steril.

[9] Scott H, Khoury J, Moore BA, Weissman S (2008). Routine anal cytology screening for anal squamous intraepithelial lesions in an urban HIV clinic. Sex Transm Dis.

[10] Turner B (1995). Medical power and social knowledge.

[11] Nidhi J, Pardaman S, Deepak G, Jyotsna K (2008). A young Asian girl with MRKH type B syndrome: a case report. International Journal of Obstetrics and Gynecology Research.

[12] Boersma-Heller J, Psych D, Schmidt H, Edmonds D (2009). Psychological distress in women with uterovaginal agenesis (Mayer-Rokitansky-Küster-Hauser Syndrome, MRKH). Psychosomatics.

[13] Mohit G, Varsha K (2012). MRKH Syndrome: psychological disturbances and suicide. J Indian Acad Forensic Med.

